# Identification of a Novel miR-122-5p/CDC25A Axis and Potential Therapeutic Targets for Chronic Myeloid Leukemia

**DOI:** 10.3390/ijms262311401

**Published:** 2025-11-25

**Authors:** Serap Ozer Yaman, Nina Petrović, Selcuk Yaman, Osman Akidan, Ahmet Cimbek, Gulsah Baycelebi, Tatjana Srdić-Rajić, Ahmad Šami, Sema Misir

**Affiliations:** 1 Department of Medical Biochemistry, Trabzon Kanuni Training and Research Hospital, Faculty of Medicine, Trabzon University, 61030 Trabzon, Turkey; 2 Laboratory for Radiobiology and Molecular Genetics, “VINČA” Institute of Nuclear Sciences-National Institute of the Republic of Serbia, University of Belgrade, 11000 Belgrade, Serbia; 3 Department of Experimental Oncology, Institute for Oncology and Radiology of Serbia, 11000 Belgrade, Serbia; tsrdic@gmail.com; 4 Department of Hematology, Mengücek Gazi Education and Research Hospital, 24100 Erzincan, Turkey; 5 Department of Internal Medicine, Trabzon Kanuni Training and Research Hospital, Faculty of Medicine, Trabzon University, 61030 Trabzon, Turkey; 6 Cellular and Molecular Radiation Oncology Laboratory, Department of Radiation Oncology, Universitaetsmedizin Mannheim, Medical Faculty Mannheim, Heidelberg University, 68167 Mannheim, Germany; 7 DKFZ-Hector Cancer Institute, University Medicine Mannheim, 68167 Mannheim, Germany; 8 Department of Biochemistry, Faculty of Pharmacy, Sivas Cumhuriyet University, 58140 Sivas, Turkey

**Keywords:** chronic myeloid leukemia, miR-122-5p, CDC25A, CDK4

## Abstract

Chronic myeloid leukemia (CML) is a myeloproliferative neoplasm characterized by uncontrolled proliferation of myeloid cells. MicroRNAs (miRNAs), small noncoding RNAs, regulate post-transcriptional gene expression by degrading target mRNAs or repressing translation. Dysregulated miRNA expression has been implicated in various malignancies, including CML, where they can function as oncogenes or tumor suppressors. This study aimed to investigate the relationship between miR-122-5p and cell division cycle 25A (CDC25A) in CML and to elucidate the regulatory mechanisms of miR-122-5p. This study integrates bioinformatics analysis with in vitro RT-qPCR validation in K562 chronic myeloid leukemia cells to explore the potential regulatory relationship between miR-122-5p and CDC25A. mRNA expression profiles were retrieved from the GSE100026 dataset in the Gene Expression Omnibus (GEO), and differentially expressed genes were identified using GEO2R. Quantitative real-time PCR (RT-qPCR) was performed to measure miR-122-5p, CDC25A, and cyclin-dependent kinase 4 (CDK4) expression levels. Bioinformatics analyses (miRNeT, miRDIP, TargetScan, BioGPS, GeneMANIA, STRING) were applied to predict molecular interactions and functional pathways. Public RNA-seq datasets and in silico tools were used to prioritize candidates; RT-qPCR in a single CML cell line (K562) provided in vitro expression validation. In K562 cells, miR-122-5p expression was significantly reduced, while CDC25A and CDK4 were markedly upregulated. Bioinformatics tools confirmed CDC25A as a potential miR-122-5p target. Functional enrichment indicated CDC25A involvement in cell cycle regulation and apoptosis. These findings suggest that miR-122-5p functions as a tumor suppressor in CML by targeting CDC25A. Modulating the miR-122-5p/CDC25A axis may provide potential molecular targets for inhibiting CML progression through regulation of cell cycle pathways. Findings are exploratory and based on bioinformatics with limited in vitro expression confirmation; functional studies are required to establish causality.

## 1. Introduction

Chronic myeloid leukemia (CML) is a myeloproliferative neoplasm originating from hematopoietic stem cells [1]. The genetic hallmark of CML is the presence of the Philadelphia chromosome (Ph) and the formation of a fusion oncogene known as BCR::ABL1 [2]. The BCR-ABL oncogene plays a role in the development of CML by leading to increased proliferation and inhibition of apoptosis through tyrosine kinase activation [3]. CML accounts for approximately 15% of leukemias and is most commonly observed between CML accounts for approximately 15% of leukemias and is most commonly observed between the ages of 40 and 60 [4]. Although tyrosine kinase inhibitors (TKIs, e.g., imatinib mesylate (IM)) have represented a significant advancement in the treatment of the disease, relapse [5] and TKI resistance remain significant challenges [6]. Therefore, a better understanding of the molecular pathology of CML and the mechanisms underlying emerging drug resistance is needed. Therefore, a better understanding of the molecular pathology of CML and the mechanisms underlying emerging drug resistance is needed to identify potential molecular target.

Recent studies have shown that microRNAs (miRNAs) play a role in CML progression and TKI resistance development based on their significant regulatory functions in cellular homeostasis [7]. miRNAs are small, noncoding RNA molecules, approximately 22 nucleotides long, critical in regulating gene expression [8]. They are involved in various biological processes, including development, differentiation, proliferation, and apoptosis. miRNAs exert their effects by binding to complementary sequences in target mRNAs, thereby regulating gene expression post-transcriptionally [9]. Accumulating evidence suggests that miRNAs play crucial roles in the development of CML. Depending on the regulated target gene, these miRNAs can function as tumor suppressors or oncogenes [10]. Therefore, identifying miRNAs and their target genes in CML is critical for understanding their roles in tumor formation and progression. Although many studies have explored the role of miRNAs, the biological functions of several miRNAs remain unclear [11].

miR-122 has the potential to act as an oncogene or tumor suppressor by targeting different genes in various cancer types. The aberrant expression of miR-122 has been reported in several cancers, including breast, lung, leukemia, and liver [12]. However, there is limited data on the expression, functions, and targets of miR-122-5p in CML.

Cell division cycle 25 A (CDC25A), a member of the CDC25 family, is a dual-specific protein phosphatase. CDC25A activates cyclin-cyclin-dependent kinase (CDK) complexes, which are critical for the transition between the G1/S and G2/M phases and the DNA damage response. Additionally, CDC25A plays significant roles in apoptosis, cell metabolism, and tumor cell metastasis [13]. Known for its oncogenic properties, CDC25A has been frequently observed in various cancer types, including lung cancer [14], breast cancer [15], and acute myeloid leukemia (AML) [16], and is closely associated with poor patient prognosis [17]. In recent years, CDC25A has garnered exceptional interest due to its identified overexpression in various cancer types [16]. However, the mechanisms underlying CDC25A overexpression remain highly complex, necessitating further research [18].

Although miR-122-5p and CDC25A have been studied in several malignancies such as hepatocellular carcinoma and acute myeloid leukemia, their coordinated regulatory relationship within the molecular context of BCR::ABL1-positive chronic myeloid leukemia has not been previously characterized. This study uniquely integrates bioinformatic prediction with in vitro expression validation to propose a disease-specific miR-122-5p/CDC25A regulatory axis potentially involved in CML pathogenesis. This study aims to determine the expression changes of miR-122-5p and CDC25A in CML, investigate their roles in its development, and explore the potential underlying mechanisms. Additionally, using in silico tools, we examined the potential role of miR-122-5p in regulating CDC25A and other CDC25A-associated genes in CML pathogenesis. Therefore, the miR-122-5p/CDC25A axis is expected to provide further insights into potential treatments for CML and improve prognosis.

## 2. Results

### 2.1. miR-122-5p Was Underexpressed in K562 Cells

**Figure 1:**
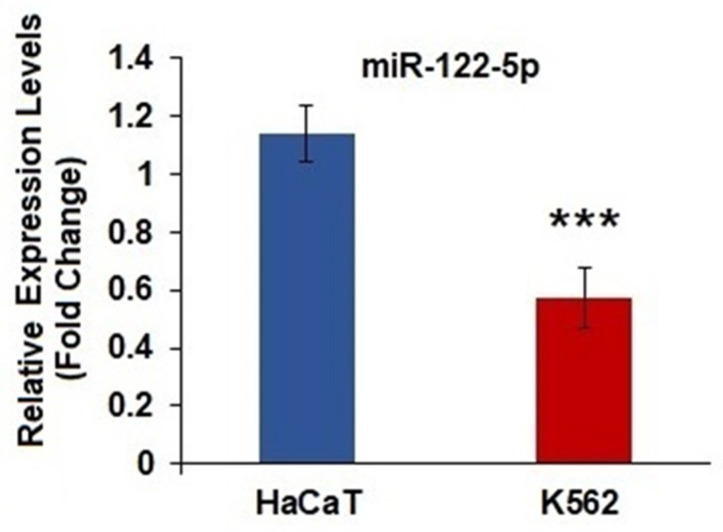
Relative miR-122-5p levels in K562 and HaCaT cells. RNU6 was used for the normalization of miRNAs. *** Represents significant data (*p* = 0.0001), compared to HaCaT cells (*n*:10).

To investigate the role of miR-122-5p in CML, miR-122-5p expression in CML cells was evaluated by RT-qPCR and compared with normal control cells. The results showed that miR-122-5p expression was significantly lower in K562 cells than in HaCaT cells (*p* = 0.0001) (Figure 1).

### 2.2. CDC25A Is a Direct Target of miR-122-5p

**Figure 2:**
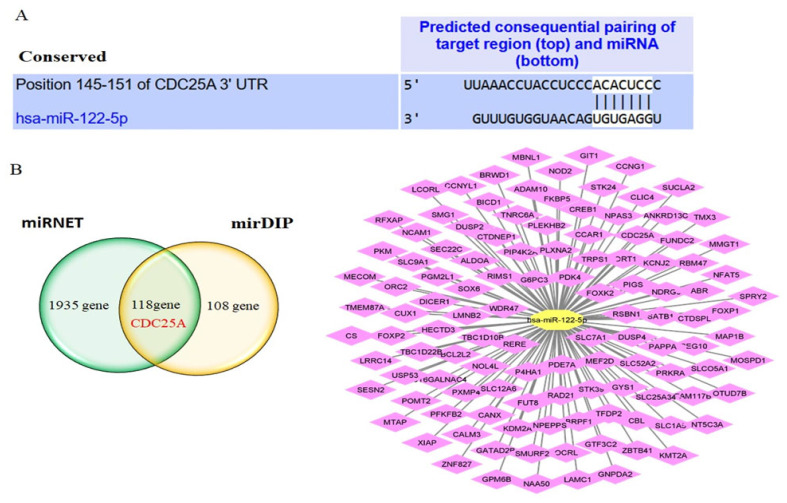
Targeting relationship between miR-122-5p and CDC25A. (**A**) The complementary sequences of CDC25A 3′UTR and miR-122-5p were presented by TargetScan. (**B**) Target genes of miR-122-5p were identified by miRNET (green colored area) and mirDIP (yellow colored area) tools. 118 overlapping genes were identified from mirNET and mirDIP tools. The miR-122-5p-targets visualization network contains 118 identified common targets. CDC25A was one of the overlapping genes in the databases selected for further analysis.

miRNAs function primarily through base pairing with sequences complementary to the seed region. To better understand the biological roles of this miR-122-5p in CML, potential target genes were predicted by miRNET and mirDIP databases. The complementary sequences of CDC25A 3ʹUTR and miR-122-5p were determined by TargetScan (Figure 2A). 2053 and 226 target genes were determined for miR-122-5p in miRNET and mirDIP, respectively (Supplementary S1). One hundred eighteen common genes from both databases were determined and visualized with Cytoscape (version number: 3.10.4) (Figure 2B). We identified genes targeted by miR-122-5p that were common between the varying mRNA profiles in CML and selected CDC25A.

### 2.3. Expression Level of CDC25A and CDK4

**Figure 3:**
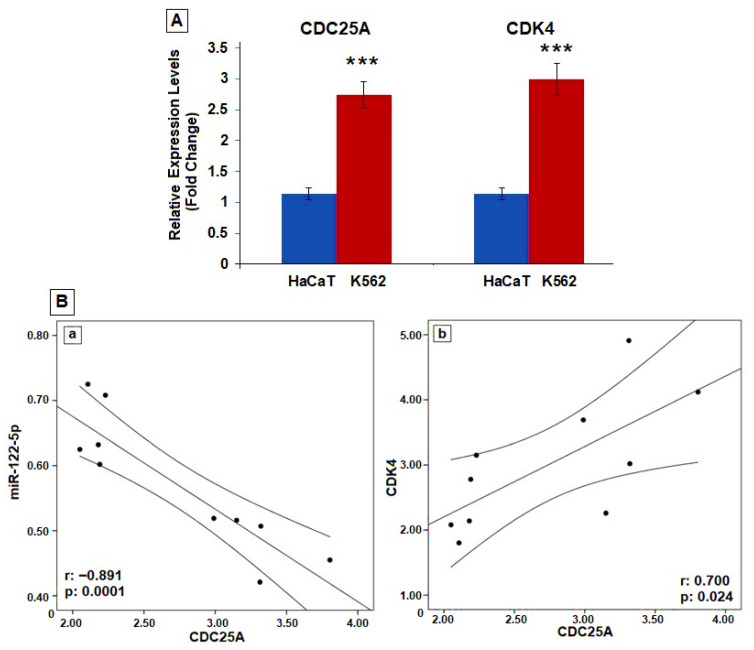
(**A**) CDC25A and CDK4 gene expression by RT-qPCR analysis in K562 and HaCaT cells. All gene expression data are presented as fold change relative to HaCaT cells. GAPDH was used for the normalization of all genes ***: (*p* = 0.0001, respectively), compared to HACAT cells. (**B**) Correlation analysis between miR-122-5p-CDC25A (**a**) and CDC25-CDK4 (**b**) expression (*n*:10).

The tissue-specific pattern of mRNA expression can give important clues about gene functions. The changed mRNA profiles between the CML and the control groups are given in Supplementary S2. Among these changed genes is CDC25A, which is also targeted by miR-122-5p. The expression of CDC25A was compared between various cancer cell lines and tissues and normal tissues using BioGPS (Supplementary S3). CDC25A expression level was high in K-562 cells. Analysis of changes in CDC25A and CDK4 gene expression in K562 and HaCaT cells revealed much higher levels of these genes in K-562 cells (Figure 3A). According to correlation analysis, a negative correlation was found between miR-122-5p expression and CDC25A expression, and a positive correlation was found between CDC25A and CDK4 (Figure 3B).

### 2.4. CDC25A Expression and Functions in Chronic Myeloproliferative Disorder

#### 2.4.1. Peripheral Blood

The PVCA suggested that CDC25A gene expression accounts for 28.4% of gene expression profile variances between the samples, while the type of diagnosed leukemia accounts for 7% of the variance. The residual factors accounted for a 64.6% variance (Supplementary S4a). Correlation heatmap for PB cohort indicated that samples in low and high tertiles had a higher correlation in gene expression profile with the samples within the same group and with the samples from medium tertile than between each other within the same gene cluster (Supplementary S4b). The DGE analysis of the PB samples cohort showed the highest number of differentially expressed genes (DEGs) when comparing high and low tertiles (n = 12,614). Comparing the median and low tertiles resulted in n = 7577 differentially expressed genes, while comparing high and medium tertiles resulted in the lowest number of DEGs (n = 2581) (Supplementary S4c).

**Figure 4:**
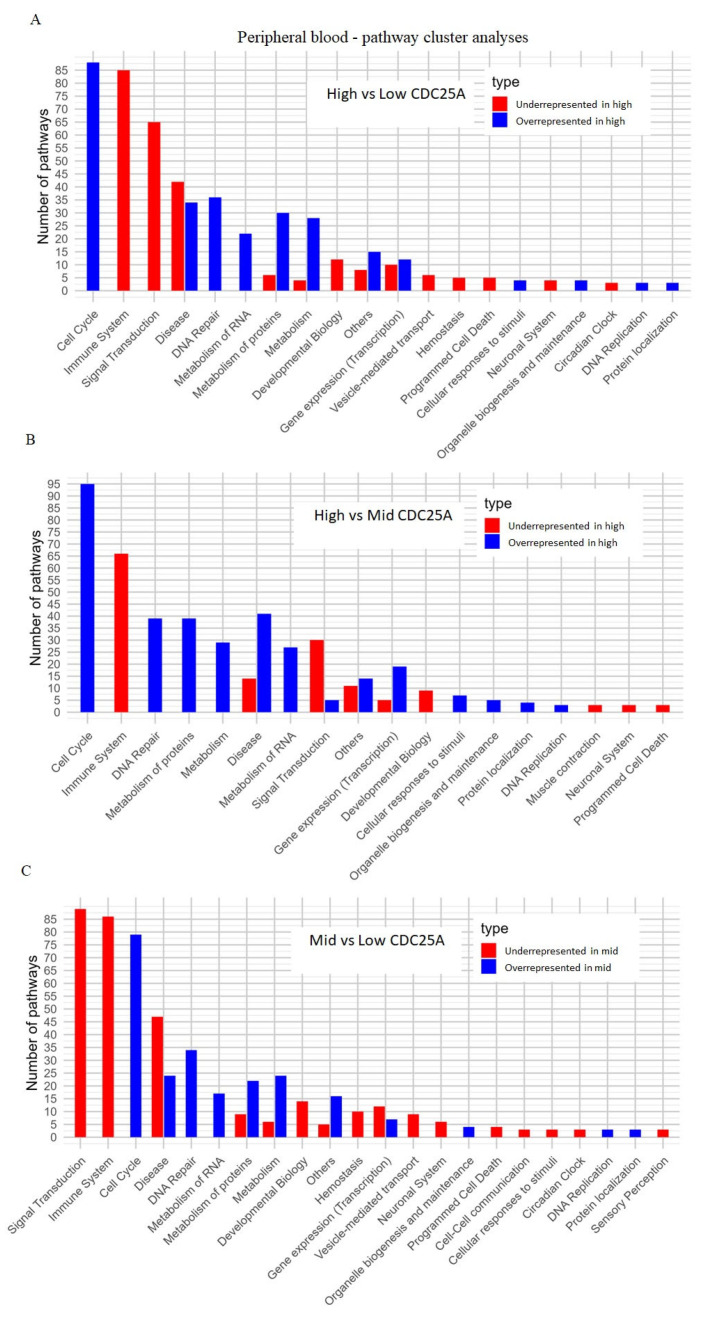
(**A**–**C**)**.** Cluster pathway analysis including the most significant pathways for each comparison—high, medium (mid) and low CDC25A expression level tertiles for peripheral blood datasets.

**Figure 5:**
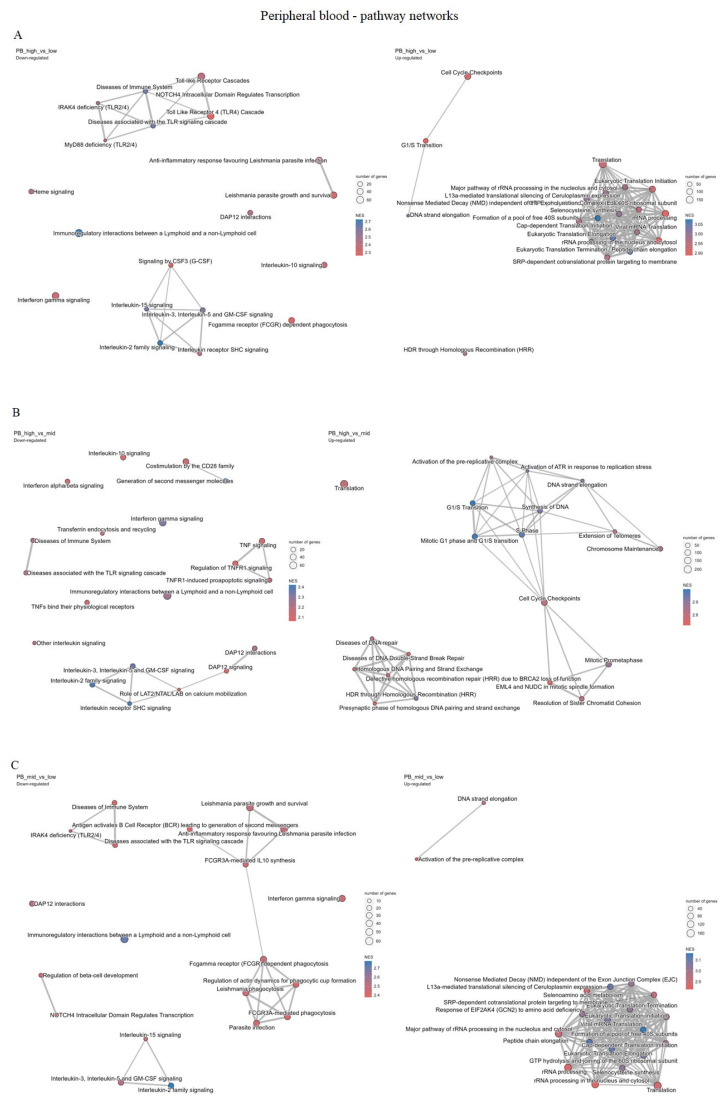
(**A**–**C**). shows pathway networks, including the most significant pathways for each comparison—high, medium (mid) and low CDC25A expression level tertiles for peripheral blood datasets.

Cluster pathway analysis suggested that samples in the high tertile compared to the samples from low tertile have overrepresented pathways related to the cell-cycle regulation and DNA repair processes while having underrepresented pathways associated with the immune system and signal transductions (Figure 4A), as well as when comparing high vs. median tertiles (Figure 4B) and when comparing median and low tertiles (Figure 4C). These results suggest that the higher the expression of CDC25A is, the more highly upregulated cell-cycle regulation and DNA repair pathways are, and the more downregulated immune system and signal transduction pathways are, on the other hand, which was also shown in Figure 5A–C (Supplementary S6).

Reactome pathway enrichment analysis visualized the top 20 up- and down-regulated pathways ranked by the normalized enrichment score (NES). Positive NES values (shown in red) indicate pathways upregulated in samples with higher CDC25A expression, whereas negative NES values (shown in blue) indicate downregulated pathways. The intensity of the color represents the magnitude of enrichment, with darker shades denoting stronger NES values. Most significantly enriched pathways were related to cell-cycle control, DNA replication, and checkpoint regulation, consistent with the functional role of CDC25A in proliferation.

#### 2.4.2. Bone Marrow

**Figure 6:**
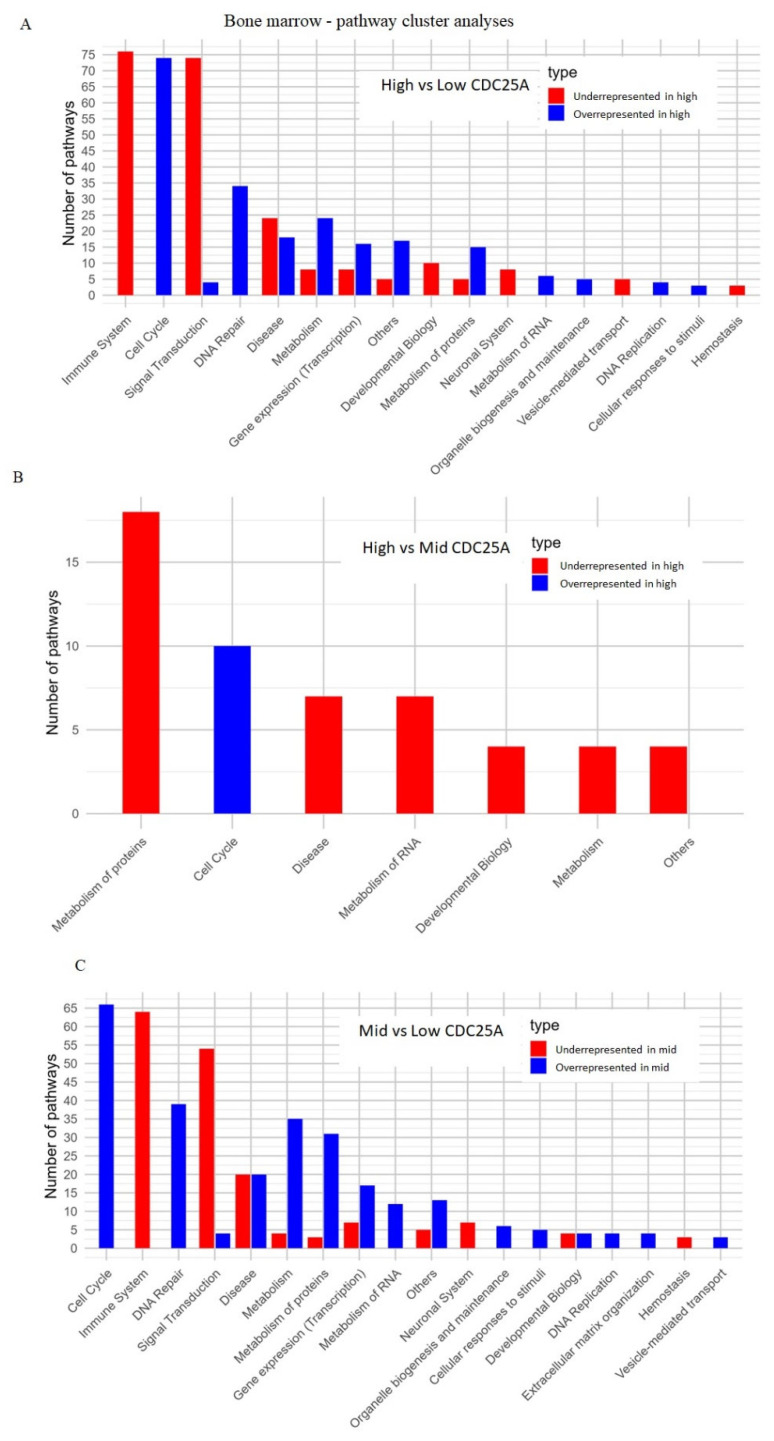
(**A**–**C**) Cluster pathway analysis, including the most significant pathways for each comparison of high, medium (mid), and low CDC25A expression level tertiles for bone marrow datasets.

**Figure 7:**
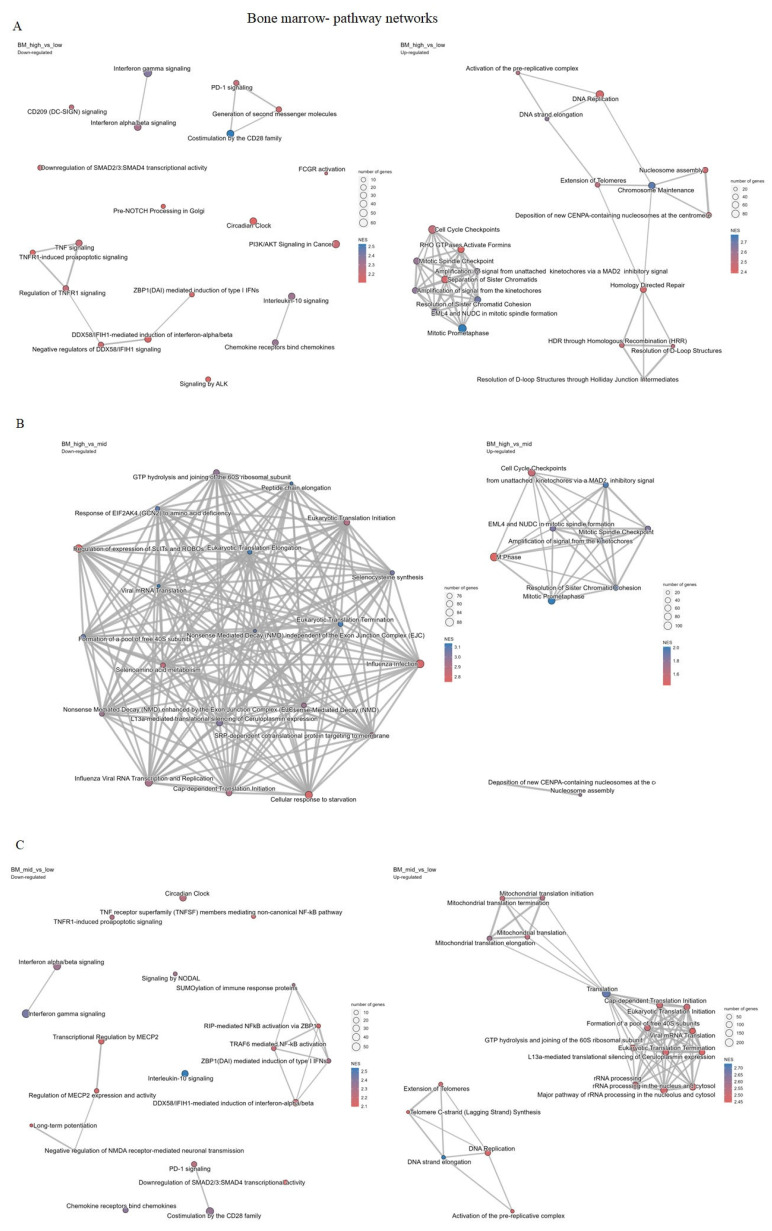
(**A**–**C**) Pathway networks, including the most significant pathways for each comparison of high, medium (mid), and low CDC25A expression level tertiles for bone marrow datasets.

Results from the analysis of the BM samples are overwhelmingly in line with the results described for the PB samples. The PVCA showed that CDC25A gene expression accounts for 35.3% of gene expression profile variances between the samples, while the type of diagnosed leukemia accounts for 13% of the variance. The residual factors accounted for 52.1% of the variance (Supplementary S5a). The same was not observed for bone marrow sample cohort where low, medium and high tertile samples were not separated into different clusters (Supplementary S5b). According to the DGE analysis of bone marrow samples, the highest number of DEGs was detected when median and low tertiles of CDC25A expression were compared (n = 767), followed by 695 differentially expressed genes between high and low tertiles, while high and medium tertiles resulted in the lowest number of DEGs (n = 13) (Supplementary S5c). These results indicate that in bone marrow samples, in a group with relatively high CDC25A expression (high and mid), not many genes and pathways may not be significantly changed, unlike between high and low and mid and low groups. In pathway cluster analysis for bone marrow sample datasets, when we compare high and low tertiles of the CDC25A gene, cell cycle, and DNA repair are most significant, while underrepresented ones are mostly associated with the immune system. When high and mid tertiles were compared, the highest underrepresented pathway was related to the metabolism of proteins, while highly overrepresented pathways were associated with the cell cycle. In mid-low comparison, cell cycle and DNA repair pathways were shown to be overrepresented, while immune system and signal transduction pathways were highly underrepresented pathways (Figure 6A–C and Figure 7A–C, Supplementary S6).

Taking all into account, higher expression CDC25A groups (mid and high, both) show similar patterns in differential gene expression and signaling pathways. Furthermore, PB and BM samples also show similar patterns, indicating that sample type, in this case, may not influence pathway and DEG analysis.

### 2.5. Interaction Network Analysis of CDC25A

**Figure 8:**
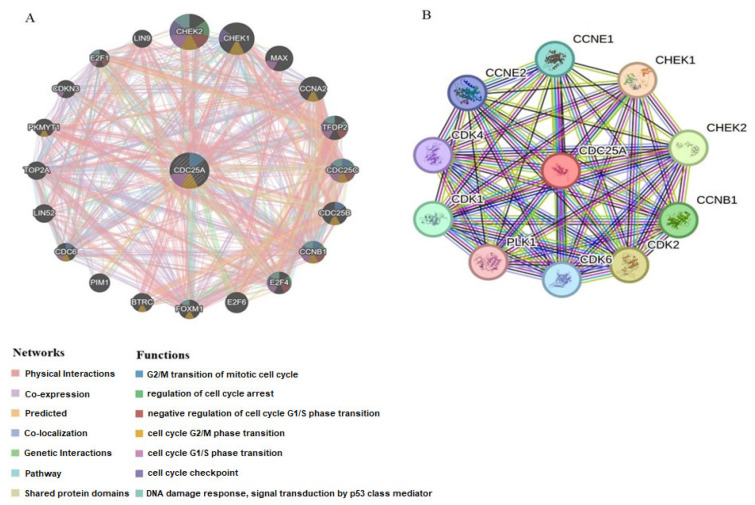
Integrated network analysis of CDC25A. (**A**) From STRING online database analysis, a total of 10 proteins were filtered into a PPI network complex. (**B**) GeneMANIA database was used to construct a gene interaction network between CDC25A and other genes.

To further uncover the biological significance of CDC25A in CML, we screened CDC25A-related proteins and genes. By using GeneMANIA and STRING, we generated the protein-protein and gene-gene interaction networks for CDC25A. Based on the gene-gene network of GeneMANIA, CDC25A interacts with 20 potential target genes (Figure 8A). Furthermore, the STRING database acquired an interaction network of the top 10 CDC25A-binding proteins (Figure 8B). These results suggested that CDC25A may function as a complex. CDC25A is specifically involved in cell cycle checkpoint and progression.

## 3. Discussion

The rapid global increase in the cancer burden highlights the urgent need to develop more effective and precise targeted therapies [19]. CML is one of the most prevalent hematologic malignancies [20], and understanding its incidence and mortality rates is crucial for advancing clinical practices [21]. Although there has been significant progress in CML treatment in recent years, effective diagnostic and therapeutic options for advanced stages of the disease remain limited [22]. Therefore, a comprehensive understanding of the molecular mechanisms of CML is essential to improve diagnostic and therapeutic approaches [11].

miRNAs are sensitive to the molecular progression of CML and play a critical role in developing resistance to TKI therapies. They also show significant potential as biomarkers [6,7]. miRNAs can act as oncogenes or tumor suppressors by directly targeting mRNAs in malignancies [23]. Consequently, miRNAs and the genes they influence represent promising therapeutic targets [7]. Recent studies have demonstrated that miRNAs may serve as potential therapeutic targets in CML, offering new possibilities for advancing treatment strategies [24,25].

This study investigates the roles of miR-122-5p and CDC25A in CML. Previous research has shown that miR-122-5p is abnormally expressed and plays significant roles in various human malignancies [26,27,28]. Studies involving hematologic cancers have indicated that miR-122-5p expression is downregulated in pediatric acute myeloid leukemia (AML) and may serve as a prognostic factor for poor outcomes. Additionally, overexpression of miR-122 in AML cell lines has been shown to inhibit cell proliferation [29]. Another study evaluated the clinical significance of miR-122 expression in response to imatinib treatment in CML patients. It was reported that miR-122 expression increases after imatinib therapy and is associated with a favorable therapeutic response [12]. Our study on K562 and HaCaT cell lines revealed lower miR-122-5p expression than controls, suggesting its potential role in CML development.

CDC25A mediates essential transitions between cell cycle stages during mitosis and is a primary target of the checkpoint process that maintains genetic stability in response to DNA damage [30]. The present study reveals a negative association between the expression levels of miR-122-5p and CDC25A in K562 cell lines, indicating a potential regulatory interaction. Our findings suggest that the overexpression of CDC25A and CDK4 markedly elevates K562 cell lines. In contrast, the expression levels of miR-122-5p diminish, implying that the overexpression of CDC25A and CDK4 may enhance the viability of these cancer cells and suppress apoptosis. The suppression of CDC25A may hinder the viability of K562 cancer cells and promote apoptosis. These findings align with prior publications. In this study, we also showed that CDC25A is one of the target genes of miR-122-5p.

Numerous studies have demonstrated that the inhibition of CDC25A can enhance the sensitivity of cancer cells to radiation, impede the survival of cancer cell colonies, and facilitate apoptosis [31,32]. The present study indicates that CDC25A is a target gene of miR-122-5p, highlighting its significant role in influencing therapeutic outcomes. Moreover, given the intricacy of miRNA target regulation, substantial efforts remain to identify and describe miR-122 targets to enhance our understanding of its biology across various tissues and malignancies. Consequently, subsequent research will ascertain if miR-122 influences chromosomal stability and other facets of tumor biology through potential targets post-DNA damage. Consequently, target genes will be identified in diverse cancers, and their therapeutic effects will be elucidated.

Bioinformatic analyses are frequently employed to study cancer progression, identify potential therapeutic targets, accurately predict cancer risk, and discover biomarkers for cancer therapies. These analyses rely on molecular or metabolomic profiling to provide critical insights into cancer biology and treatment strategies [33,34]. In this study, bioinformatic analyses were conducted to identify the target mRNAs of miR-122-5p. Using miRNET and mirDIP, we identified 118 common target mRNAs, and CCDC25A was selected for further investigation due to its altered expression in CML. Binding sites for miR-122-5p within the 3′ untranslated region (3′UTR) of CDC25A were identified through TargetScan. Recent studies have increasingly focused on inducing apoptosis and halting the cell cycle as strategies for cancer treatment. Previous studies have implicated miR-122-5p and CDC25A in a range of cancers, including hepatocellular, breast, and acute myeloid leukemia [35,36]. However, no study to date has examined their potential co-regulation in chronic myeloid leukemia. The present work extends current knowledge by identifying a negative correlation between miR-122-5p and CDC25A expression in K562 cells and by demonstrating that CDC25A-associated pathways are enriched in CML-related datasets. This context-specific analysis provides an initial framework for exploring a novel miRNA-mediated regulatory axis in CML. Previous studies have demonstrated that CDC25A overexpression contributes to leukemogenesis through deregulation of the G1/S checkpoint and enhancement of DNA replication stress. Fernández-Vidal et al. reported that aberrant CDC25A activation drives oncogenic transformation by promoting cyclin-dependent kinase activity [16]. Similarly, Shen & Huang and Lara-Chica et al. confirmed its role in maintaining proliferative signaling and genomic instability in hematologic malignancies [13,18]. In this context, our findings suggesting an inverse relationship between miR-122-5p and CDC25A expression in CML are consistent with these mechanistic observations. CDC25A is one of the key regulators of the cell cycle [37]. Due to its proto-oncogenic properties, overexpression of CDC25A has been observed in various malignancies, including Non-Hodgkin Lymphoma and esophageal, gastric, lung, thyroid, head, and neck cancers [38]. CDC25A plays a role in tumor initiation and progression and is associated with poor prognosis, making it a potential target for cancer therapy [18,19]. Many miRNAs, such as miR-16 [39], let-7 [40], miR-15a [41], miR-449a/b [42], miR-21 [43], miR-125b [44], miR-424 [45], miR-449 [46], and miR-483 [47], have been identified as regulators of CDC25A, highlighting the importance of miRNA-mediated suppression in cell cycle control. Another study reported that miR-122-5p enhances the radiosensitivity of cervical cancer cells by targeting CDC25A [31]. According to our findings, K-562 cells had higher levels of CDC25A expression than control cells. Additionally, a negative connection between miR-122-5p and CDC25A was found using correlation analysis, suggesting that the two have a negative relationship.

We further investigated the effects of the miR-122-5p/CDC25A axis in greater detail. An interaction network analysis was conducted to identify the proteins and genes associated with CDC25A in the development of CML and to elucidate the potential mechanisms of its action. According to results derived from GeneMANIA and STRING databases, CDC25A is mainly associated with cyclin-dependent kinases (CDK1, 2, 4, 6), the E2F transcription factor family (E2F1, E2F4, E2F6), cyclins (Cyclin E and Cyclin A), and checkpoint kinases (Chk1 and Chk2). These genes and proteins are closely linked to cell cycle regulation and apoptosis. In addition to the well-established role in modulating the G1/S transition through effects on the CCNE–CDK2 complex, CDC25A has also been identified as a key regulator in the early stages of the cell cycle by targeting CDK4/6 [43]. Furthermore, CDC25A plays a role in G2 phase progression by promoting the dephosphorylation of CDK1 and activating the Cyclin B1/CDK1 complex [18,48]. Another study suggested that CDC25A could remove the inhibitory phosphorylation of CDK4, acting as a positive regulator of CDK4 [18]. In contrast to control cells, we found that K562 cells in our study expressed more CDK4. Correlation analysis showed a positive association between CDC25A and CDK4, suggesting a connected role in cell cycle regulation. In the pathway cluster analysis for bone marrow and peripheral blood datasets, the higher the CDC25A expression, the more regulated the cell cycle regulation and DNA repair pathways are. Although transcriptomic validation utilized predominantly Philadelphia-negative myeloproliferative samples, these analyses were designed to identify conserved CDC25A-associated signatures within myeloid neoplasms. Therefore, our conclusions regarding CML pathogenesis are exploratory and require further validation in BCR::ABL1-positive patient samples.

The choice of HaCaT cells as a control line represents a methodological limitation, as these cells are not of hematopoietic origin. They were employed in this exploratory phase primarily for their stability and reproducibility as a non-malignant reference. Nonetheless, future investigations using normal hematopoietic cells (PBMCs or CD34^+^ progenitors) and primary CML patient samples are essential to validate whether the observed inverse expression pattern between miR-122-5p and CDC25A is conserved in the hematopoietic environment.

Given these results, it is suggested that miR-122-5p acts as a tumor suppressor. miRNA replacement treatment might provide a strategy to restore the lost contributions of miR-122-5p because of its lower expression in CML. The use of miR-122-5p mimics could inhibit cell proliferation in CML. miR-122-5p negatively regulates CDC25A expression, modulates DNA replication signaling pathways by contributing to the miR-122-5p/CDC25A network, and can promote apoptosis. miR-122-5p and CDC25A are thought to be targets for the treatment of CML. Additionally, CDC25A inhibitors are promising as therapeutic targets for cancer treatment.

Although further experimental and clinical validation is required, a novel miRNA-mRNA regulatory axis has been identified, which could enhance the understanding of the molecular mechanisms underlying CML development. These target molecules hold potential as both prognostic biomarkers and therapeutic targets, paving the way for improved diagnostic and treatment strategies. Although our study provides preliminary evidence supporting a potential regulatory association between miR-122-5p and CDC25A, we acknowledge that functional assays are necessary to confirm causality. Future experiments involving miR-122-5p mimic/inhibitor transfection and CDC25A silencing or overexpression will be performed to verify their direct effects on cell cycle progression, proliferation, and apoptosis in CML models. While our results revealed an inverse correlation between miR-122-5p and CDC25A expression in K562 cells, these data alone are insufficient to establish a direct regulatory relationship. The study was designed as an exploratory analysis combining bioinformatics prediction and gene expression validation. Functional assays such as miR-122-5p mimic/inhibitor transfection, CDC25A siRNA knockdown, and overexpression studies are essential to confirm causality and to determine whether miR-122-5p directly regulates CDC25A-mediated cell-cycle and apoptosis pathways. Future experiments will focus on these gain- and loss-of-function approaches to substantiate the proposed miR-122-5p/CDC25A regulatory axis in CML. Another limitation of this work is the small sample size of the GSE100026 dataset, which may reduce the statistical robustness of differential expression analysis. The study should therefore be viewed as exploratory rather than confirmatory. Nevertheless, the integration of multiple bioinformatics resources allowed cross-validation of predicted miRNA–mRNA interactions and identification of consistent targets such as CDC25A. Future studies will validate these findings using larger, independent CML cohorts and additional transcriptomic datasets.

## 4. Materials and Methods

### 4.1. Differentially Expressed Genes (DEGs) and Data Processing

The National Center for Biotechnology Information (NCBI) Gene Expression Omnibus (GEO) (http://www.ncbi.nlm.nih.gov/geo, accessed on 3 October 2025) provided the dataset that we used in this investigation [49]. The GEO database, which was deposited by Li et al., provided the mRNA expression profile data for GSE100026 based on the GPL18573 platform (Illumina NextSeq 500, San Diego, CA, USA) used in this investigation [22]. The data from GSE100026 included changes in mRNA profiles of peripheral blood mononuclear cells from five patients in chronic phase (CP), five patients in blast crisis (BC) phase of chronic myeloid leukemia, and five healthy volunteers. GEO2R (www.ncbi.nlm.nih.gov/geo/geo2r, accessed on 3 October 2025) was used to identify differentially expressed mRNAs between CML and the control group [50]. The GSE100026 dataset includes peripheral-blood mononuclear cell profiles from five chronic-phase, five blast-crisis CML patients, and five healthy controls. Although the small sample size restricts statistical power, the dataset was selected for its well-annotated sequencing quality and representation of distinct CML disease phases. To enhance prediction robustness, multiple miRNA-target databases (miRNet, miRDIP, TargetScan, STRING, GeneMANIA) were employed, and only genes overlapping across at least two platforms were considered. This cross-validation approach minimizes prediction bias. A stringent logFC ≥ 6 threshold was applied to highlight markedly dysregulated transcripts, acknowledging that this criterion may exclude moderately altered genes. Many experimental datasets are included in GEO2R, and false favorable rates vary using an adjusted *p* value (adj. *p*). The adjusted *p*-value cut-off point for selecting mRNAs was set at *p* < 0.05 and llogFCl > 6. We preferred the logFC threshold value to be 6 and above because the fold value of the target molecule is above 6.

### 4.2. Prediction of miRNA Targets

We employed the miRNET (Upgraded R to version 4.1.3) (https://www.mirnet.ca/, accessed on 1 June 2025) [51] and mirDIP (https://ophid.utoronto.ca/mirDIP/, accessed on 1 June 2025) [52,53] databases to determine the potential target gene of hsa-miR-122-5p. In miRNeT, miRNA module was selected to identify genes targeted by miR-122-5p, human (homo sapiens) organism was identified. miRBase ID was chosen as the ID type, and gene modules were selected as targets. While identifying miR-122-5p targets using the mirDIP database, miR-122-5p was added to the miRNA modules section, and the score class section was chosen as very high. Firstly, the common target genes obtained from miRNET and mirDIP were determined. Genes in common with miRNET and mirDIP overlapped with the gene changes in GSE100026, and the common ones were selected. We used Cytoscape software to build a miRNA-target visual network. Also, using TargetScan (https://www.targetscan.org/vert_80/, accessed on 1 June 2025), the CDC25A 3′UTR and miR-122-5p complementary sequences were determined [54].

### 4.3. CDC25A Gene Expression Analysis in K562

The BioGPS database (http://biogps.org, accessed on 1 June 2025) was used to analyze the expression profiles of CDC25A in different cancer and paired normal cell lines [55,56]. The gene modulene CDC25A was entered and was selected in the resulting gene list. In the new tab that opened, the gene expression module was selected in the Add a plugin section.

For in silico validation, publicly available RNA-seq data from the Philadelphia-Negative Neutrophilic Leukemias (OHSU-CNL) project were used. These datasets, derived from peripheral blood and bone marrow of patients with chronic myeloproliferative disorders, provide one of the few accessible transcriptomic cohorts suitable for evaluating CDC25A-associated pathways in myeloid neoplasms.

#### CDC25A Gene Expression Analysis and Functions in Chronic Myeloproliferative Disorder

This study analyzed samples from the Philadelphia-Negative Neutrophilic Leukemias (OHSU-CNL) project, collected from Chronic Myeloproliferative Disorders patients [57]. From the TCGA repository, 66 RNA-seq datasets were downloaded from peripheral blood (PB) samples and 10 RNA-seq datasets from bone marrow (BM) samples. PB and BM samples were analyzed separately. Samples were divided into low, medium, and high tertiles for the CDC25A expression levels, according to the transcript per million (TPM) values. For the downstream analysis, raw counts for the selected samples were downloaded from the TCGA repository. Low expressed genes were filtered out with EdgeR package (version number: 3.14. 0), followed by data normalization with voom function from limma package in R. Differential gene expression (DGE) analysis was performed with limma package, while enrichment pathway analysis was performed with ReactomePA package in R. Diagnosed leukemia subtypes were not considered an experimental factor, except for the Principal Variance Component Analysis (PVCA). PVCA was performed to estimate the impact of CDC25A gene expression levels on general gene expression profile variations between the samples. Correlation heatmaps were generated to show similarities in expression profiles between samples. Differential gene expression (DGE) and enrichment pathway analysis were performed by comparing the tertiles against each other. Pathway cluster analysis was performed by grouping the pathways according to their top-level pathway in the Rectome hierarchy. Pathway networks were generated for the top 20 upregulated and downregulated significant pathways in each comparison.

### 4.4. Gene and Protein Network of CDC25A

We constructed a protein and gene interaction network to elucidate the interactions between miRNA and its target gene. Protein/protein (PPI) and interacting genes network analysis of CDC25A were examined using the Search Tool for the Retrieval of Interacting Genes/Proteins (STRING) database (http://www.string-db.org/) [58]. Another online resource, GeneMANIA (http://genemania.org/), is an online predictive analytics tool that looks at target genes’ functional associations, co-expression, co-localization, domain protein similarities, and protein and genetic linkages [59]. The GeneMANIA database was also used to network CDC25A-associated target genes.

### 4.5. Cell Culture

CML cell line (K562, CCL-243) and human keratinocyte cells (HaCaT, PCS-200-011) cells from American Type Culture Collection (Rockville, MD, USA) were cultured in Dulbecco’s Modified Eagle Medium (DMEM) supplemented with 10% fetal bovine serum (FBS), and penicillin (192 U/mL), streptomycin (200 mg/mL) under the conditions of relative humidity, 5% CO_2_, and 37 °C. Following 80% confluence, cells were harvested for further examination and testing. HaCaT cells were chosen as the non-malignant reference model owing to their genetic stability and reproducible miRNA/mRNA expression characteristics. This allowed us to assess the baseline regulatory effect of miR-122-5p on CDC25A independent of hematopoietic lineage-specific transcriptional programs. HaCaT cells (human keratinocytes) were selected as a genetically stable, non-malignant control model due to their well-characterized phenotype and reproducible baseline expression profile. The use of HaCaT cells enabled the evaluation of miR-122-5p/CDC25A expression dynamics under standardized, lineage-independent conditions. However, we acknowledge that hematopoietic cells, such as PBMCs or CD34^+^ progenitors, would represent a more physiologically relevant control. Future studies will incorporate these cell types to confirm the lineage-specific regulation of the miR-122-5p/CDC25A axis in CML.

### 4.6. RNA Extraction and Quantitative Real-Time PCR (RT-qPCR) for CDC25A, CDK4

The total RNA was extracted from all cells collected by using the RNA Isolation Kit (GeneAll, RiboEx, Cat: 301-001, Seoul, Republic of Korea) according to the manufacturer’s recommendations. cDNA synthesis using a cDNA Synthesis Kit with Rnase Inh. (High Capacity) (A.B.T., Cat: C03-01-05, Ankara, Türkiye), according to manufacturer’s protocols. Reaction mixtures were incubated in at 25 °C, 10 min; 37 °C, 120 min; and 85 °C, 5 min.

RT-qPCR was performed using A.B.T. 2X SYBR Green Mastermix (A.B.T., Cat: Q03-01-05, Ankara, Türkiye), according to manufacturer’s protocols. About 20 mL PCR included 4 mL cDNA product, 1 mL (10 μM) forward primer, 1 mL (10 μM) reverse primer, 1 mL ROX, 3 mL sterile water, and 10 mL (2X) SYBR master mix. The following primers were used: CDC25A-F 5′-TTC CTC TTT TTA CAC CCC AGT CA-3′, CDC25A R 5′-TCG GTT GTC AAG GTT TGT AGT TC-3′, CDK4-F5′-GTG TAT GGG GCC GTA GGA AC-3′, CDK4-R 5′-CAG TCG CCT CAG TAA AGC CA-3′, GAPDH-F 5′-TGA CTT CAA CAG CGA CAC CCA-3′, GAPDH-R 5′-CAC CCT GTT GCT GTA GCC AAA-3′ (Oligomer Biotechnology, Çankaya, Ankara, Türkiye). CDC25A and CDK4 expression levels were normalized to the amount of GAPDH in the same sample. The reaction mixtures were incubated in at 95 °C for 5 min, followed by 40 cycles of 95 °C for 15 s, and 60 °C for 60 s. Ct (threshold cycle) values were determined by the 2^−ΔΔCt^ method [60].

### 4.7. miRNA Extraction and RT-qPCR Analysis

The expression of miR-122-5p was assessed using a real-time polymerase chain reaction (PCR) technique in accordance with the manufacturer’s instructions. According to manufacturer specifications, cDNA was synthesized using (High Capacity) (A.B.T., Cat: C03-01-05, Ankara, Türkiye). Target-specific pre-amplification used diluted cDNA (1:4) with miScript primer (miR-122-5p) and miScript PreAMP PCR Kit (Qiagen, Cat: 331452, Hilden, Germany). U6 snRNA served as the internal control. For RT-qPCR analysis, GAPDH was used as the internal reference for mRNA quantification, and U6 snRNA served as the endogenous control for miRNA analysis. Relative gene expression levels were calculated using the 2^−ΔΔCt^ method. Each reaction was run in technical triplicates, and mean Ct values were used for statistical analysis. 5 µL of cDNA template were mixed with SYBER Green Master Mix (Qiagen, Cat: 218073, Hilden, Germany) and with miScript primer assays (Qiagen, Cat: 218300 Hilden, Germany) to make a 20 µL mixture. This was then added to a custom 96-well miScript miRNA PCR plate that had forward and reverse miRNA-specific primer for miR-122-5p. LightCycler 480 system (Roche, Basel, Switzerland) was used to conduct real-time PCR procedures. The conditions for real-time PCR were as follows: 95 °C for 15 min, succeeded by 40 cycles of 94 °C for 15 s, 55 °C for 30 s, and 70 °C for 34 s, 1 cycle at 95 °C for 1 s, 60 °C for 1 min and 1 cycle (cooling) 95 °C. The cycles needed for the fluorescence signal to exceed the threshold (background noise level) were computed to ascertain the cycle threshold (CT) in real-time PCR. The CT values of SNORD61 were subtracted from the CT values of the target miRNAs to ascertain the ΔCt values of the miRNAs. ΔCt values are inversely correlated with miRNA expression levels. Consequently, reduced ΔCt values correlate with elevated miRNA expression. Each sample was analyzed in triplicate. Using the comparative threshold cycle 2^−ΔΔCq^ method, miRNA expression levels were calculated after being normalized to the external reference miR-122-5p and the internal reference U6 snRNA (U6 snRNA-F-5′-CTCGCTTCGGCAGCACA-3′, U6 snRNA-R-AACGCTTCACGAATTTGCGT-3′), [60].

### 4.8. Statistical Analysis

Statistical analyses were performed using IBM SPSS Statistics for Windows (version 23.0; IBM Corp., Armonk, NY, USA). The conformity of variables to normal distribution was determined using the Kolmogorov–Smirnov test. Differences in the expression of miR-122-5p, CDC25A, and CDK4 genes between K562 and HaCaT cells were evaluated using the independent samples t-test. In light of the skewness of the data distribution, Pearson correlation analysis was applied to assess the relationships between miR-122-5p and CDC25A genes. In K562 cells, Pearson correlation analysis was evaluated separately between miR-122-5p-CDC25A and CDC25A-CDK4 expression results. Statistical significance was determined as *p* < 0.05. All experiments were performed in 10 repetitions.

## 5. Conclusions

In this study, we successfully constructed a miRNA-mRNA regulatory network related to CML. Our findings suggest that miR-122-5p may regulate CDC25A expression through complementary base pairing. By reducing the expression of CDC25A, miR-122-5p could potentially control the cell cycle and restrict cellular proliferation. Although further experimental and clinical validation is required, these insights could contribute to a deeper understanding of the molecular mechanisms underlying CML and highlight potential therapeutic targets. In conclusion, the current findings should be interpreted as preliminary and correlative. Although miR-122-5p and CDC25A show opposing expression patterns in CML cells, direct mechanistic validation is required. Subsequent studies involving functional modulation of miR-122-5p and CDC25A will be necessary to confirm their causal relationship and biological significance in CML proliferation and survival. This exploratory study identifies a potential inverse association between miR-122-5p and CDC25A expression in chronic myeloid leukemia. These results suggest that miR-122-5p may participate in pathways influencing cell-cycle regulation, but further functional and mechanistic validation is required to establish causality. Therefore, the findings should be viewed as hypothesis-generating and form a foundation for future studies exploring miR-122-5p/CDC25A axis dynamics in CML pathogenesis.

## Data Availability

The data supporting this study’s findings are available on request from the corresponding author.

## Supplementary Materials

The following supporting information can be downloaded at: https://www.mdpi.com/article/10.3390/ijms262311401/s1.

## Abbreviations

The following abbreviations are used in this manuscript:

AML	Acute myeloid leukemia
CDC25A	Cell division cycle 25A
CML	Chronic myeloid leukemia
CDK	cyclin-cyclin-dependent kinase
CDK4	Cyclin-dependent kinase 4
CT	Cycle threshold
DGE	Differential gene expression
DMEM	Dulbecco’s Modified Eagle Medium
FBS	Fetal bovine serum
GEO	Gene Expression Omnibus
HaCaT	Human keratinocyte cells
IM	Imatinib mesylate
miRNAs	MicroRNAs
Ph	Philadelphia chromosome
PVCA	Principal Variance Component Analysis
RT-qPCR	Quantitative real-time PCR
STRING	Search Tool for the Retrieval of Interacting Gene
TKI	tyrosine kinase inhibitors
