# Supplementary material for: Identification of a Novel miR-122-5p/CDC25A Axis and Potential Therapeutic Targets for Chronic Myeloid Leukemia
Source: Int J Mol Sci. 2025 Nov 25;26(23):11401. doi: 10.3390/ijms262311401 (PMC12692635; doi:10.3390/ijms262311401)
Supplement: Supplementary file 1 [file ijms-26-11401-s001.zip › Supplementary 1.pdf]

Supplementary1. 2053 and 226 target genes were determined for miR-122-5p in miRNET and mirDIP
GSE100026

GeneID	padj	pvalue	lfcSE	log2Fold Change	Symbol	Description
3575	8.77e-75	5.32e-79	0.337	-6.35	IL7R	interleukin 7 receptor
4603	6.19e-39	4.13e-42	0.45	-6.11	MYBL1	MYB proto-oncogene like 1
1524	2.05e-37	1.49e-40	0.457	-6.09	CX3CR1	C-X3-C motif chemokine receptor 1
8320	2.75e-34	3.67e-37	0.485	-6.18	EOMES	eomesodermin
4318	2.56e-29	5.74e-32	0.761	8.96	MMP9	matrix metallopeptidase 9
115352	4.06e-28	1.11e-30	0.538	-6.19	FCRL3	Fc receptor like 3
162394	7.12e-27	2.25e-29	0.54	-6.08	SLFN5	schlafen family member 5
6590	9.03e-27	2.90e-29	0.803	9.01	SLPI	secretory leukocyte peptidase inhibitor
147841	2.75e-26	9.35e-29	0.591	6.58	SPC24	SPC24 component of NDC80 kinetochore complex
112267973	1.27e-25	4.48e-28	0.605	-6.65	LOC112267973	replaced by ID 387357
285596	2.51e-25	9.12e-28	0.603	-6.58	FAM153A	family with sequence similarity 153 member A
56265	2.66e-25	9.83e-28	0.605	6.61	CPXM1	carboxypeptidase X, M14 family member 1
109729184	3.21e-25	1.29e-27	0.819	8.91	SLC12A5-AS1	SLC12A5 and MMP9 antisense RNA 1
401124	1.20e-24	5.03e-27	0.583	-6.28	DTHD1	death domain containing 1
55859	8.24e-23	4.14e-25	0.733	7.59	BEX1	brain expressed X-linked 1
332	2.52e-22	1.36e-24	0.634	6.49	BIRC5	baculoviral IAP repeat containing 5
931	2.16e-21	1.33e-23	0.621	-6.21	MS4A1	membrane spanning 4-domains A1
5553	3.58e-21	2.26e-23	0.976	9.72	PRG2	proteoglycan 2, pro eosinophil major basic protein
4353	1.01e-20	6.71e-23	0.952	9.38	MPO	myeloperoxidase
202134	3.53e-20	2.42e-22	0.695	-6.76	FAM153B	family with sequence similarity 153 member B
128239	5.55e-20	3.97e-22	0.662	6.4	IQGAP3	IQ motif containing GTPase activating protein 3
3240	1.17e-19	8.99e-22	0.774	7.42	HP	haptoglobin
8645	1.45e-19	1.14e-21	0.691	6.61	KCNK5	potassium two pore domain channel subfamily K member 5
10112	1.62e-19	1.29e-21	0.652	6.23	KIF20A	kinesin family member 20A
10321	2.12e-18	1.89e-20	0.767	7.11	CRISP3	cysteine rich secretory protein 3

GeneID	padj	pvalue	lfcSE	log2Fold Change	Symbol	Description
57126	2.38e-18	2.14e-20	0.905	8.38	CD177	CD177 molecule
1511	5.64e-18	5.40e-20	1.102	10.09	CTSG	cathepsin G
306	6.23e-18	6.01e-20	0.71	6.5	ANXA3	annexin A3
1991	1.02e-17	1.00e-19	1.077	9.79	ELANE	elastase, neutrophil expressed
116159	1.06e-17	1.05e-19	0.669	6.08	CYYR1	cysteine and tyrosine rich 1
6037	1.18e-17	1.18e-19	0.742	6.73	RNASE3	ribonuclease A family member 3
10024	1.67e-17	1.72e-19	0.715	6.45	TROAP	trophinin associated protein
200132	2.00e-17	2.09e-19	0.772	6.96	DYNLT5	dynein light chain Tctex-type family member 5
81624	3.13e-17	3.31e-19	0.682	6.11	DIAPH3	diaphanous related formin 3
8288	3.44e-17	3.65e-19	0.968	8.66	EPX	eosinophil peroxidase
3624	7.57e-17	8.40e-19	0.71	6.29	INHBA	inhibin subunit beta A
100507612	1.27e-16	1.44e-18	0.768	-6.76	LINC00402	long intergenic non-protein coding RNA 402
8993	1.87e-16	2.16e-18	0.957	8.37	PGLYRP1	peptidoglycan recognition protein 1
8302	2.20e-16	2.58e-18	0.806	-7.04	KLRC4	killer cell lectin like receptor C4
11065	3.09e-16	3.66e-18	0.74	6.43	UBE2C	ubiquitin conjugating enzyme E2 C
3934	7.28e-16	9.01e-18	0.908	7.79	LCN2	lipocalin 2
1669	1.27e-15	1.61e-17	1.086	9.25	DEFA4	defensin alpha 4
671	1.90e-15	2.47e-17	0.927	7.85	BPI	bactericidal permeability increasing protein
8447	2.77e-15	3.73e-17	0.842	7.09	DOC2B	double C2 domain beta
993	3.42e-15	4.69e-17	0.765	6.42	CDC25A	cell division cycle 25A
10661	6.12e-15	8.70e-17	0.897	7.46	KLF1	KLF transcription factor 1
1235	6.41e-15	9.25e-17	0.853	-7.09	CCR6	C-C motif chemokine receptor 6
4057	7.21e-15	1.05e-16	0.929	7.71	LTF	lactotransferrin
1116	1.27e-14	1.92e-16	0.839	6.9	CHI3L1	chitinase 3 like 1
