# Supplementary material for: Identification of a Novel miR-122-5p/CDC25A Axis and Potential Therapeutic Targets for Chronic Myeloid Leukemia
Source: Int J Mol Sci. 2025 Nov 25;26(23):11401. doi: 10.3390/ijms262311401 (PMC12692635; doi:10.3390/ijms262311401)

4a. PVCA analysis for 66 peripheral blood sample datasets

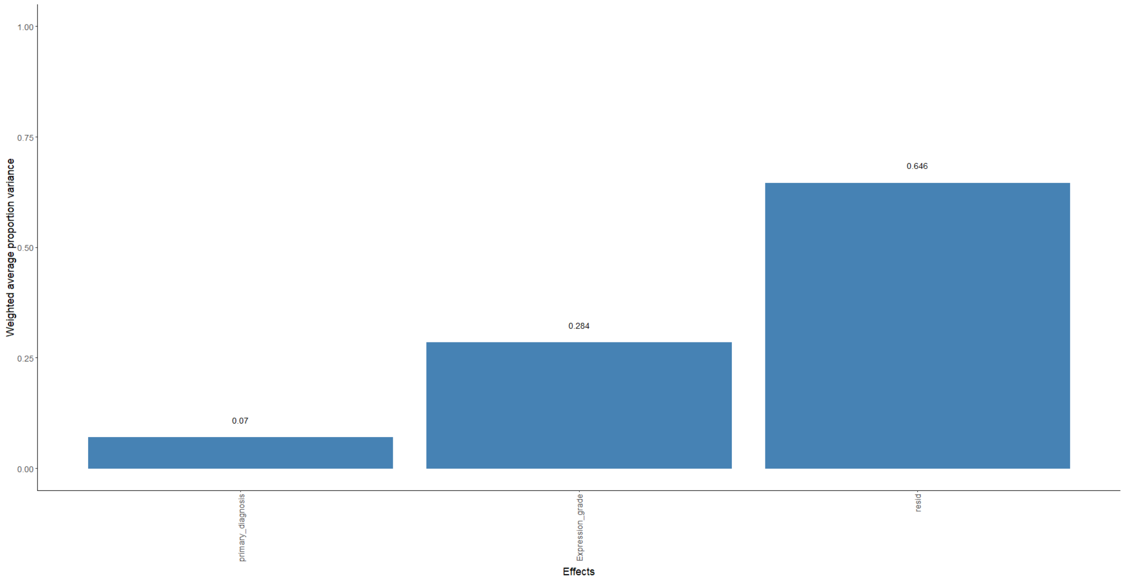

4b. Correlation heatmap for peripheral blood sample datasets, n = 66

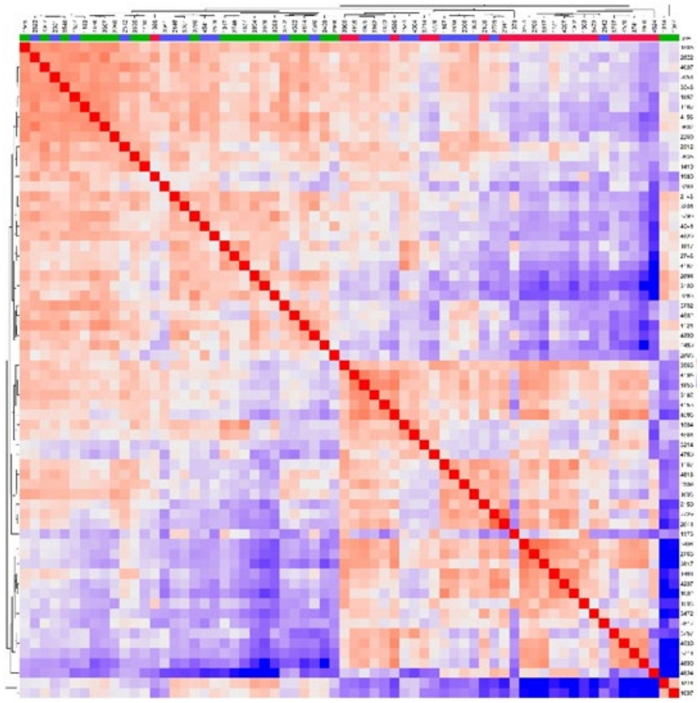

4c) Number of differentially expressed genes between high, medium (mid) and low CDC25A expression level tertiles for peripheral bloosd sample datasets

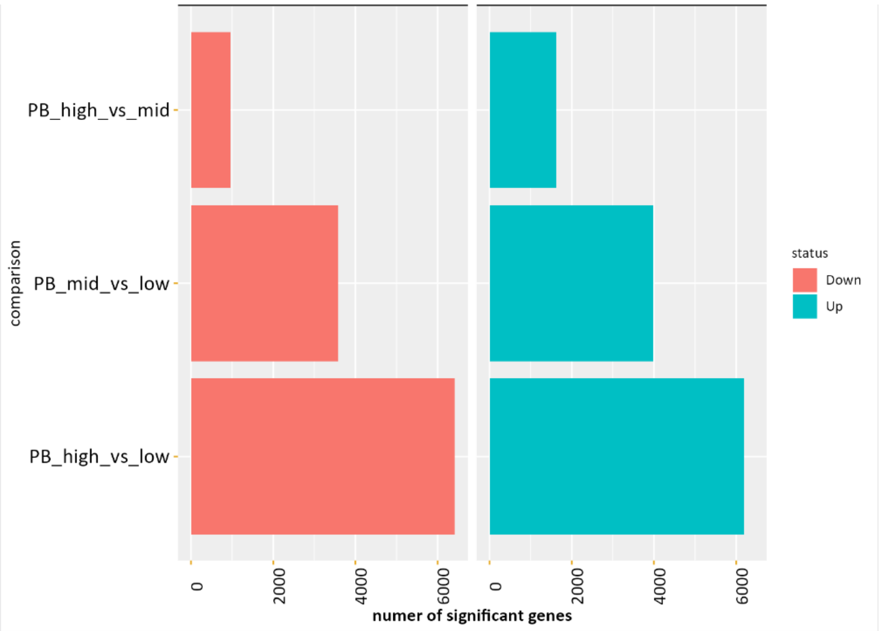

Supplement: Supplementary file 1 [file ijms-26-11401-s001.zip › Supplementary 4a, 4b, 4c .pdf]
