# Supplementary material for: Identification of a Novel miR-122-5p/CDC25A Axis and Potential Therapeutic Targets for Chronic Myeloid Leukemia
Source: Int J Mol Sci. 2025 Nov 25;26(23):11401. doi: 10.3390/ijms262311401 (PMC12692635; doi:10.3390/ijms262311401)
Supplement: Supplementary file 1 [file ijms-26-11401-s001.zip › Supplementary 5a, 5b, 5c .pdf]

A) PVCA analysis for 10 bone marrow sample datasets

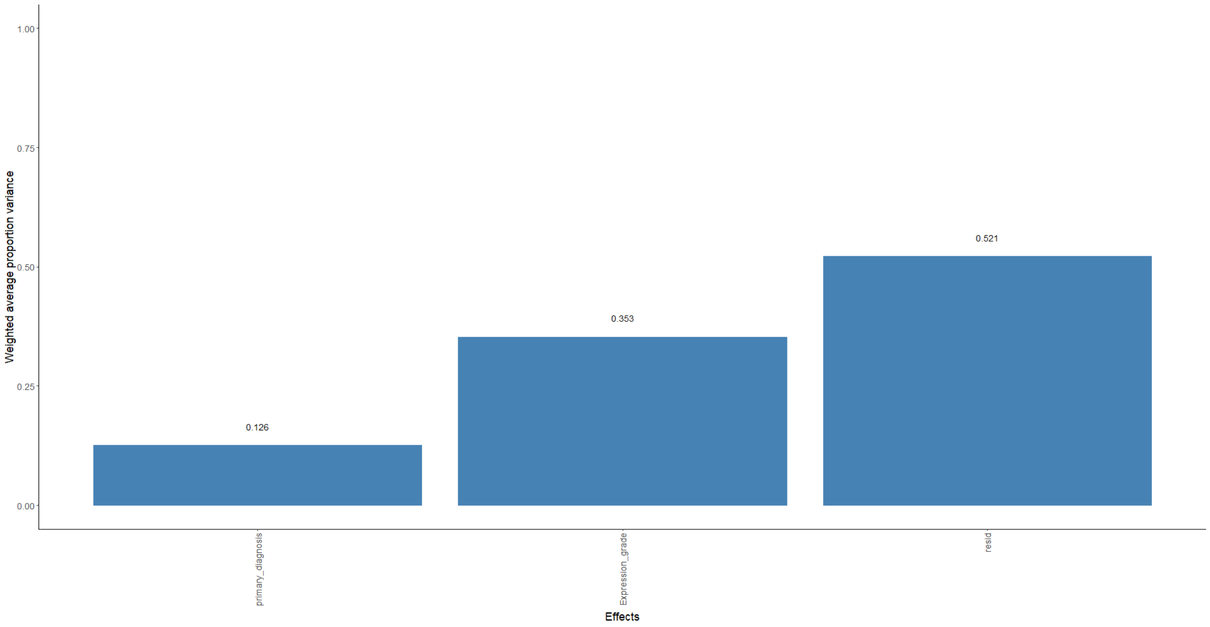

B) Correlation heatmap for bone marrow sample datasets, n =10

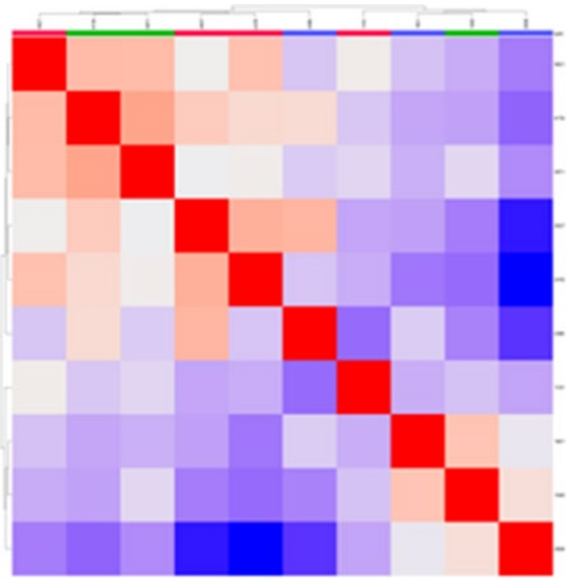

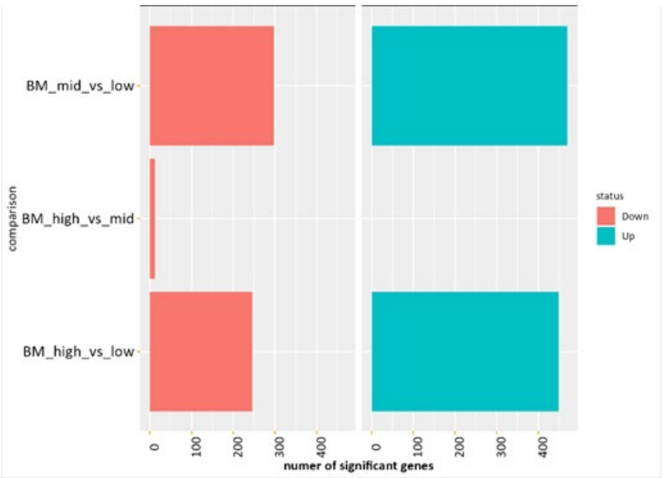

C) Number of differentially expressed genes between high, medium (mid) and low CDC25A expression level tertiles in bone marrow sample datasets
