# Supplementary figures and images for: Identification of a Novel miR-122-5p/CDC25A Axis and Potential Therapeutic Targets for Chronic Myeloid Leukemia
Source: Int J Mol Sci. 2025 Nov 25;26(23):11401. doi: 10.3390/ijms262311401 (PMC12692635; doi:10.3390/ijms262311401)

Supplementary 3. CDC25A expression levels in various cancer cell lines and tissues from BioGPS.

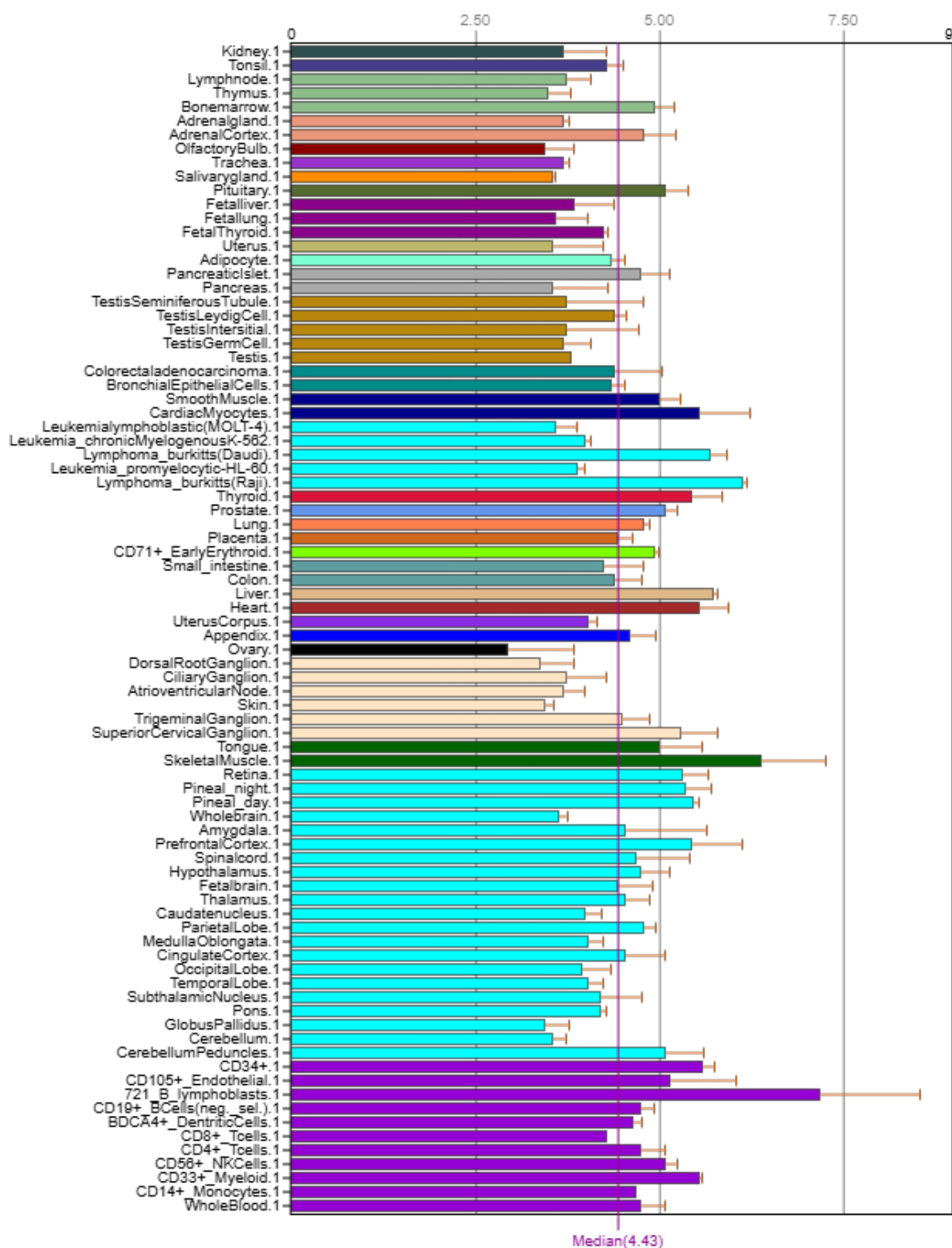

Supplement: Supplementary file 1 [file ijms-26-11401-s001.zip › Supplementary 3.pdf]
